# Effects of Meglumine Antimoniate Treatment on Cytokine Production in a Patient with Mucosal Leishmaniasis and Chagas Diseases Co-Infection

**DOI:** 10.3390/tropicalmed5020069

**Published:** 2020-05-02

**Authors:** Karine Rezende-Oliveira, Cesar Gómez-Hernández, Marcos Vinícius da Silva, Rafael Faria de Oliveira, Juliana Reis Machado, Luciana de Almeida Silva Teixeira, Lúcio Roberto Cançado Castellano, Dalmo Correia, Virmondes Rodrigues

**Affiliations:** 1 Laboratory of Biomedical Sciences, Federal University of Uberlândia, Ituiutaba, Minas Gerais CEP 38304-402, Brazil; karinerezende@ufu.br; 2 Laboratory of Immunology, Federal University of Triângulo Mineiro, Uberaba, Minas Gerais CEP 38025-180, Brazil; virmondes.junior@uftm.edu.br; 3 Laboratory of Parasitology, Federal University of Triângulo Mineiro, Uberaba, Minas Gerais CEP 38025-180, Brazil; marcos.silva@uftm.edu.br; 4 Laboratory of Clinical Analysis, Professional Education Center, Federal University of Triângulo Mineiro, Uberaba, Minas Gerais CEP 38025-404, Brazil; rafael.oliveira@uftm.edu.br; 5 Discipline of General Pathology, Federal University of Triângulo Mineiro, Uberaba, Minas Gerais CEP 38025-200, Brazil; juliana.machado@uftm.edu.br; 6 Infectious Diseases Division, Federal University of Triângulo Mineiro, Uberaba, Minas Gerais CEP 38025-180, Brazil; lalmeidas@terra.com.br (L.d.A.S.T.); dalmo@mednet.com.br (D.C.); 7 Human Immunology Research and Education Group, Technical School of Health of Federal University of Paraíba, João Pessoa, Paraíba CEP 58059-900, Brazil; luciocastellano@gmail.com

**Keywords:** mucosal leishmaniasis, Chagas disease, co-infection, antimoniate therapy

## Abstract

The influence of antimoniate treatment on specific anti-protozoan T-cell responses was evaluated in a 48-year-old male patient diagnosed with mucosal leishmaniasis and Chagas disease infection. Before and after treatment, PBMC (peripheral blood mononuclear cells) were cultured in the absence or presence of *Leishmania braziliensis* or *Trypanosoma cruzi* live parasites, their soluble antigens, or PHA (phytohaemagglutinin). Cytokines were measured and Treg (T regulatory) cell percentages were quantified. Before treatment, PBMC were able to produce higher amounts of TNF-α, IL-6 (Interleukin-6), and IL-10 (Interleukin-10) but lower amounts of IL-12 (Interleukin-12) in response to culture stimulation. However, after treatment, there was a down-modulation of TNF-α, IL-6, and IL-10 cytokines but an up-modulation in IL-12 production. PBMC had the ability to produce TNF-α only against live parasites or PHA. There was an overall decrease of circulating Treg cells after treatment. In mixed Leishmaniasis and Chagas disease infection, treatment with antimoniate could modulate immune responses toward a more protective profile to both diseases.

## 1. Introduction

American Tegumentary Leishmaniasis (ATL) and Chagas disease (CD) are protozoan infections caused by *Leishmania sp.* and *Trypanosoma cruzi*, respectively. These neglected diseases occur in the tropics with an overlapping distribution of endemic areas, especially in South America [1]. Both diseases show diverse clinical presentations varying from localized cutaneous form to mucosal manifestations (ML) for ATL and from asymptomatic individuals to patients with digestive abnormalities to patients with severe heart failure for CD [1]. Immune response against CD is characterized by pro-inflammatory cytokine production, while in ATL, significant anti-inflammatory cytokine production has been observed. Furthermore, a particular role developed by T regulatory cells (Tregs) has been considered. Treatments for both diseases are very complex and some effects of the first-choice drugs remain unclear. Drugs indicated for CD treatment are nifurtimox and benznidazole, both presenting an average cure rate from 80% in acute disease to less than 20% in chronic infections [2]. In the case of ML, the pentavalent antimonial drugs still are the first option, although they present important side effects and variable efficacy. Amphotericin B and pentamidine are also suggested; however, their cost and the toxicity observed in clinical studies restrict their use in ATL treatment [3].

There are few reports in the literature providing evidences of patients with suspected or confirmed mixed infections [4,5,6,7,8,9]. Usually, these reports explore the cross-reactivity among parasite’s antigens used in serodiagnostic by ELISA (Enzyme-Linked Immunosorbent Assay) kits or the establishment of a more specific molecular tool rather than patients’ clinical status. However, the immunological responses of patients with mixed *L. braziliensis* and *T. cruzi* infections still need to be evaluated. In this context, we aimed to evaluate the influence of treatment with meglumine antimoniate on the specific anti-protozoan T-cell response in a case of mixed mucosal leishmaniasis and Chagas disease infection.

## 2. Case Report

**Figure 1:**
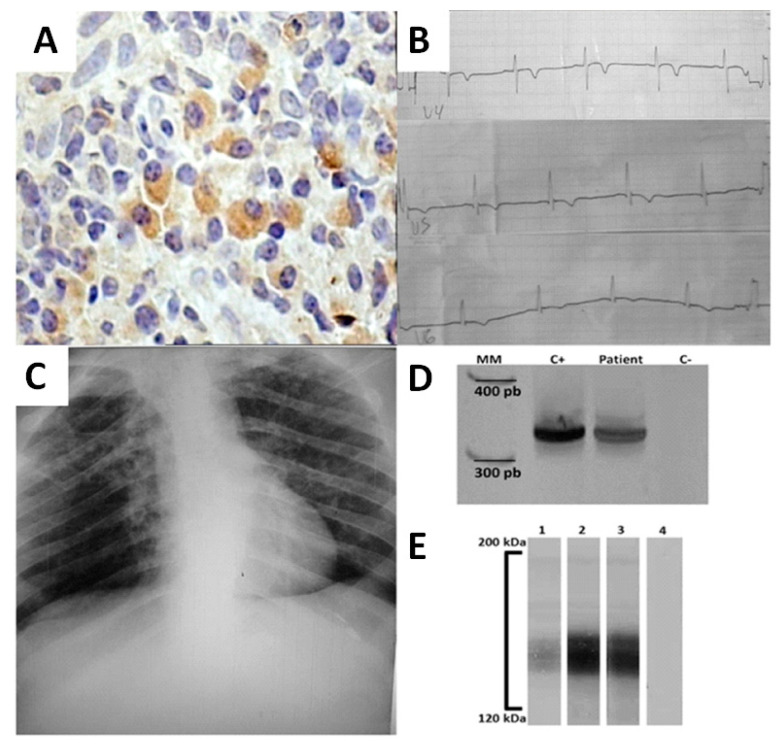
Clinical data and laboratorial findings of a patient with Chagas disease and Leishmaniasis co-infection. (**A**) Immunohistochemistry for the detection of Leishmania’s antigens in nasal septum biopsy. Brown areas indicate the presence of *Leishmania sp.* antigens (**B**) Electrocardiographic alterations showing V4, V5, and V6 derivations with T-wave inversion indicating chronic chagasic cardiopathy. (**C**) Chest radiography. (**D**) Molecular detection of *T. cruzi* DNA using specific primers 121–122 by PCR. Lines MM- 100 bp molecular marker; C+ positive control; patient-patient’s sample; C− negative control. (**E**) TESA-blot positive for *T. cruzi* before and after treatment of patient. Lines 1—positive control; 2—patient’s sample before treatment; 3—patient’s sample after treatment; 4—negative control.

A 48-year-old male was admitted to the Clinical Hospital of Federal University of Triângulo Mineiro, with a four-month progressive mucosal lesion on his septum. The patient reported itching and occasional epistaxis. At the time of admission, physical examination revealed a septal perforation and a roundish scar on his left leg as a consequence of a previous ulcerative lesion spontaneously healed 3 years ago. Immunohistochemical analysis of the nasal biopsy was positive for *Leishmania* amastigotes, whereas histology was negative for *Mycobacterium sp.* and fungi (Figure 1A). The patient presented normal blood pressure and heart rate. Chest and abdominal radiography (Figure 1C) as well as abdominal ultrasound were normal. Electrocardiography (ECG) revealed a T-wave inversion on V4, V5, V6, and diffuse ventricular repolarization abnormalities (Figure 1B). Transesophageal echocardiography demonstrated a left ventricular ejection fraction (EF) = 50% and shortening fraction of 26%, right and left atrial and right ventricular normal dimensions. Increased left ventricular size with decreased systolic performance and diffuse hypokinesis. As a result of the patient’s origin from an endemic area for Chagas disease and due to his altered ECG, serology for *T. cruzi* was performed and was positive in all tests: indirect hemagglutination assay, indirect immunofluorescence, and TESA-blot (BioMérieux, Brazil) is an immunoblotting assay that uses secreted and excreted trypomastigote antigens (Figure 1E). In order to discriminate from a serological cross-reactivity with *Leishmania* antigens, the molecular detection of *T. cruzi* DNA was performed by PCR (Figure 1D) using the following primers that amplify a 330 bp fragment: 121 (5′-AAA TAA TGT ACG GGK GAG ATG CAT GA-3′) and 122 (5′-GGT TCG ATT GGG GTT GGT GTA ATA TA-3′) [10]. Serology for HIV was negative. The patient was diagnosed with mucosal leishmaniasis (ML) and Chagas disease co-infection, chronic fase with cardiac form (functional class II of the New York Heart Association). The patient’s treatment was in accordance with standard Brazilian Ministry of Health clinical practice. Meglumine antimoniate (Glucantime^®^) treatment was started for 30 days, being 20 mg Sb + 5/kg/day for 13 days and 15 mg Sb + 5/kg/day for 17 days due to detectable hepatotoxicity. The patient remained hospitalized for 40 days and was discharged and sent to the Plastic Surgery Division for evaluation and outpatient follow up.

**Figure 2:**
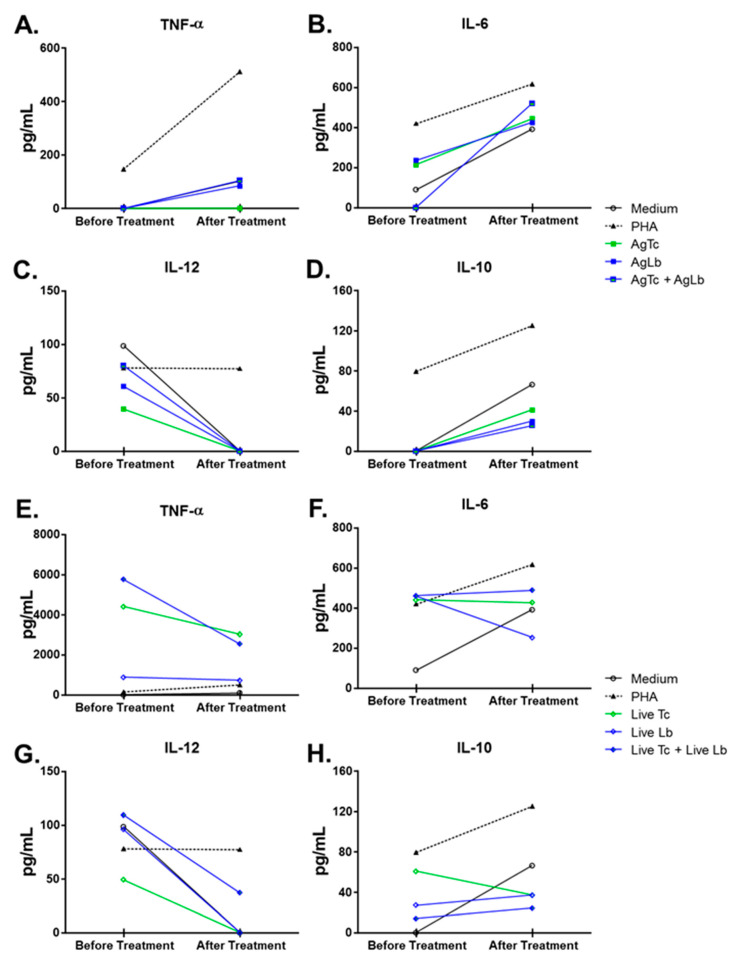
Cytokine’s production by peripheral blood mononuclear cells (PBMC) stimulated in vitro before and after patient’s treatment with meglumine antimoniate. Graph (**A**–**D**) depicts the level of (**A**) TNF-α; (**B**) IL-6; (**C**) IL-10, and (**D**) IL-12 detected by ELISA in culture supernatants before and after treatment. Graphs (**E**–**H**) depict the fold induction capacity of antigens and live parasites on cytokine production over basal stimulation (medium alone). Open bars represent results before treatment and full bars represent results after treatment.

Venous blood was collected in two different time periods: (1) at the moment of patient’s admission, just after clinical evaluation and before any treatment regimen and (2) at the end of specific treatment for mucocutaneous leishmaniasis (40-day period of Glucantime^®^ regimen). In both periods, peripheral blood mononuclear cells (PBMC) were separated using Ficoll-Paque^TM^ Plus gradient (GE Health Care, Uppsala, Sweden) and cultured in RPMI 1640 (GIBCO, Grand Island, NY, USA) in 8 different culture conditions: medium alone, 2 ug/mL Phytohaemagglutinin (Sigma, St. Louis, MO, USA), 3:1 *T cruzi* (Y strain) or 3:1 *L. braziliensis* (Lb2904 strain) live parasites, as well as 5 ug/mL *T. cruzi* and 5 ug/mL *L. braziliensis* soluble proteins, in a 5% CO_2_ atmosphere at 37 ℃ for 24 h, as described elsewhere [11,12]. The supernatants were used for cytokine quantification, IL-6, IL-10, TNF-α and IL-12, by ELISA assay as previously described [11,13]. Briefly, for each cytokine quantification, 96-well flat-bottomed polystyrene microtiter ELISA plates (Costar, Corning, NY) were coated with indicated monoclonal antibody 1 μg/mL (BD Pharmingen, San Diego, CA, USA). Non-reactive sites in the wells were blocked with 2% BSA (bovine serum albumin) in coating buffer. Culture supernatants were added at 100 μL/well and incubated for 2 h at room temperature. Horseradish peroxidase conjugated anti-cytokine monoclonal antibodies (BD Pharmingen, San Diego, CA, USA) were added at 1 μg/mL. After, TMB (tetramethylbenzidine) substrate solution (BD Pharmingen, San Diego, CA, USA) was used for assay revelation, which was read on a microplate reader at 750 nm (Bio-Rad Microplate reader-Benchmark, Hercules, CA, USA), and sample concentrations were determined by simple regression over a standard curve and values were obtained in pg/mL (Figure 2A−D). The results expressed on Figure 2E,H were elaborated considering the equation:

$$\frac{\left(\mathrm{cytokine}\;\mathrm{levels}\;\mathrm{in}\;\mathrm{stimulated}\;\mathrm{cells}\;-\;\mathrm{cytokine}\;\mathrm{levels}\;\mathrm{in}\;\mathrm{medium}\;\mathrm{alone}\right)}{\mathrm{cytokine}\;\mathrm{levels}\;\mathrm{in}\;\mathrm{medium}\;\mathrm{alone}}$$

**Figure 3:**
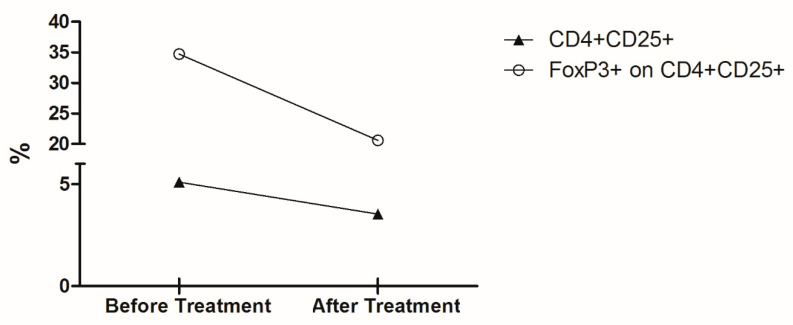
Percentage of circulating cells with T regulatory phenotype before and after treatment. Graph depicts the ex vivo percentage of CD4^+^CD25^+^ T cells (▲) and the percentage of Foxp3^+^ cells on CD4^+^CD25^+^ T cells subpopulation (◯) from the patient’s PBMC collected before and after treatment with meglumine antimoniate.

Another fraction of isolated PBMC was subjected to ex vivo immunophenotyping. Cells were suspended in 100 µL of Hanks’ balanced salt solution (Sigma, St. Louis, MO, USA) at a final concentration of 5 × 10^5^ cells/mL and incubated with 5 µL of fluorochrome-conjugated antibodies (BD Pharmingen, San Diego, CA, USA) against the following surface markers: CD4-PE-Cy7 (clone RPA-T4) and CD25-FITC (clone PC61). For intracellular staining, cells were permeabilized with BD Cytofix/Cytoperm^TM^ Plus (BD Biosciences) and then incubated with 10 µl of the FoxP3-PE (clone 259D/C7) antibody. Multiparameter flow cytometry was performed using a FACScalibur flow cytometer (Becton Dickinson, Mountain View, CA, USA) compensated with single fluorochromes. Data were analyzed using Cell Quest Pro software (Becton Dickinson). Dead cells were omitted by side scatter/forward scatter (SSC/FSC) gating, and isotype-matched control antibodies were used to determine background levels of staining (Figure 3).

## 3. Results and Discussion

The occurrence of an overlapping distribution of ATL and CD in endemic areas would be detrimental for a large number of patients to be diagnosed with mixed infections [1]. However, few reports on the literature demonstrated these two diseases affecting the same group of patients at the same time [4,5,6,7,8,9]. All these studies were drowning to promote accurate diagnostic tests that are capable of eliminating serological cross-reactivity between *Leishmania sp.* and *T. cruzi*. Despite their relevancy to the field, some important issues still need attention. The present study aimed to deal with one of the missing aspects by evaluating the potential effects of meglumine antimoniate treatment on cellular immune responses of a patient with ATL/CD co-infection. Soon after the case definition but still before any treatment, it was decided to analyze the patient’s immunological response by evaluating the production of some cytokines (TNF-α, IL-6, IL-10, and IL-12) that might have an impact in both ML and CD pathogenesis. The blood sample was collected before the patient’s treatment with meglumine antimoniate and just before patient discharge; another venous blood sample was collected and processed in order to evaluate the possible immunomodulatory effects of treatment.

The capacity of cells to respond to the various stimuli was affected by the treatment with meglumine antimoniate; there seemed to be TNF-α increasing levels when cells were stimulated with AgLb and AgLb + Lc and decreasing production with stimuli of live Lc and live Lc + Lb. In contrast, stimulation with live parasites induced high levels of TNF-α and antigens (Figure 2A). IL6 increased in production when stimulated with parasite antigens and decreased when stimulated with live Lb (Figure 2B). There were decreasing levels of IL-12 with any stimuli (Figure 2C). The IL10 had increased production except with live Tc stimuli. Considering the importance of circulating Treg cells in patients with one of each disease when presented alone [13,14,15], it was decided to evaluate the effect of the meglumine antimoniate treatment over Treg cell counts in this patient, presenting a mixed infection. There was an overall decrease in the number of CD4^+^CD25^High+^ T cells as well as in the percentage of Foxp3^+^ cells within this population after treatment.

In general, treatment with meglumine antimoniate seemed not to induce changes in TNF-α levels, thus increasing the production of IL-6 and IL-10 and decreasing the levels of IL-12 (Figure 2A−D). The capacity of cells to respond to the various stimuli was also observed when the basal levels were subtracted, considering the cultured cells in medium alone (Figure 2E,H). Before treatment, patient’s peripheral blood cells were stimulated by live parasites, and their soluble proteins or PHA produced higher levels of TNF-α, IL-6, and IL-10 but lower IL-12 cytokines in comparison to culture medium alone (basal production). However, after treatment, there was a remarkable change in the ability of cells to respond to stimuli; this was observed by the down-modulation of TNF-α, IL-6, and IL-10 cytokines but the up-modulation in IL-12 production, which recovered the ability to achieve baseline levels. Cytokine levels were quite often detected at equal or below the amount measured in a medium condition alone. Interestingly, cultured cells still had the ability to produce TNF-α only against live parasites (Live-Tc and Live-Lb) or phytohaemagglutinin (PHA), but not against parasite antigens (Figure 2E). Considering the importance of circulating Treg cells in patients with one of each disease when presented alone [13,14,15], it was decided to evaluate the effect of the meglumine antimoniate treatment over Treg cell counts in this patient, presenting a mixed infection. There was an overall decrease in the number of CD4^+^CD25^High+^ T cells as well as in the percentage of Foxp3^+^ cells within this population after treatment.

There is strong evidence showing that an unbalanced production of inflammatory/modulatory cytokines is crucial to tissue damage and worse prognosis in both ML and CD. Studies conducted in ATL patients with localized cutaneous lesions (LCL) had shown that treatment with meglumine antimoniate decreased the IL-4 and IL-10 production by antigen-stimulated PBMC [11,13,16,17]. The reactive CD4^+^ T cells still produced significant IL-10 levels, even after the treatment of ATL patients [17]. In addition, it has been shown that genetic polymorphisms in the promoter region of the *il10* gene induced elevated amounts of IL-10 independently of IFN-γ (Interferon-γ) production in patients with LCL [13]. Increased IFN-γ and TNF-α production was observed in both CD4^+^ and CD8^+^ T cells post-chemotherapy [17]. Higher levels of specific anti-*Leishmania* CD4^+^ and CD8^+^ T cells had been observed in patients with ML in comparison to individuals with LCL. The IFN-γ production did not change in ML after treatment and was always higher than that observed in patients with CL [16]. Again, a functional polymorphism in the IFN-γ gene induced lower INF-γ production in ATL patients. However, the allele frequencies studied could not distinguish between healthy control subjects and ATL patients or between CL and ML forms of the disease, nor could it be associated to disease susceptibility or worse prognosis [18]. On the other hand, polymorphism in the intron region of the TNF gene was associated to elevated cytokine levels in sera from ATL patients in comparison to healthy subjects. In addition, it was associated with a higher risk for mucosal involvement in infected patients [19]. These data indicate that strong TNF-α response in treated patients might predict unfavorable outcomes in the evolution from CL to ML forms of the disease, whereas a down-modulation in IL-4 and IL-10 production would play an important role in infection control and disease better prognosis. Our results reinforces data regarding the role of meglumine antimoniate treatment decreasing TNF-α and IL-10 production in the ML/CD patient, which would be beneficial to clinical cure and disease remission. Considering the Treg cells, it has been demonstrated that this population recovered from the infected skin of LCL patients produces IL-10 and exerts an immunomodulatory phenotype that is capable of inhibiting the proliferation of CD4+ effector T cells [14]. Patients with active LCL lesions presented a higher percentage of circulating CD4^+^CD25^+^Foxp3^+^ T cells in comparison to both patients with healed lesions and endemic area inhabitants with a resistant phenotype [13]. The decrease in the percentage of circulating CD4^+^CD25^+^Foxp3^+^ T cells observed in the case related here indicates that successful healing would be partially dependent on the decrease of this cell population induced by the meglumine antimoniate chemotherapy. Previous work has shown that treatment with meglumine antimoniate interferes with phagocytosis in monocytes from LCL patients [20]. The authors reported that meglumine antimoniate increased IFN-γ serum levels, while it up-regulated the phagocytic function of monocytes in association with the increase in production of TNF-α but not IL-10 by these cells. Together with our results, it suggests a strong immunomodulatory effect of meglumine antimoniate in distinct cell subsets.

Chagasic patients with an indeterminate form or presenting less aggressive cardiomyopathy have an increase in IL-10 and IL-17 production as well as in the number and in the suppressive function of CD4^+^CD25^high^Foxp3^+^ Treg cells than patients with severe cardiomyopathy. Moreover, it was shown that Treg cells from cardiac patients were able to produce elevated levels of IL-6, IFN-γ, and TNF-α and are unable to modulate the TNF-α and IFN-γ production from leukocytes [15,21]. Interestingly, it has been observed that the proper TNF-α itself induces IL-10 production in human monocytes as a negative feedback loop [22]. Higher cytokine production together with genetic polymorphisms in the promoter region of the TNF-α gene was associated with human infection with *T. cruzi* [23]. An in situ analysis of cytokine production in the heart tissues of subjects who had died during the chronic phase of Chagas disease suggested a pivotal role of TNF-α and IFN-γ-producing cells in promoting heart failure and fibrosis [24,25]. One might infer that circulating Treg cells would bring protection to indeterminate patients. Thus, the fact that these cells are not enough to dampen the inflammatory response in more severe cardiac forms of the disease suggests that diminishing the levels of Treg cells in cardiac patients would not be immediately associated with a worse prognosis. In this way, our results seem to be promising by the fact that treatment decreased the percentage of circulating CD4^+^CD25^+^Foxp3^+^ T cells, concomitantly to the decreased ability of leukocytes to produce TNF-α, IL-6, and IL-10 in response to live parasites or their antigens. Live trypomastigotes of the same *T. cruzi* Y strain used in our study were able to induce TNF-α and IL-12 but not IL-10 production in PBMC of CD seronegative healthy individuals [12]. This suggests an important *in vivo* clonal expansion of parasite-specific IL-10-producing T cells.

The administration of antimoniate may induce cardiac alterations that are observed in ECG [26]. This would be detrimental to patients already presenting altered ECG. However, in the patient studied here, who was diagnosed with Chagas disease, the treatment with meglumine antimoniate did not promote any enhancement on cardiac commitment already existent. Additional studies with a larger number of patients are needed to evaluate a possible role of meglumine antimoniate in chagasic cardiomyopathy. 

## 4. Conclusions 

The results presented here highlight the importance of a complete clinical and laboratorial investigation of patients coming from geographical regions that present overlapping endemic areas for parasitic diseases. In case of a mixed Leishmaniasis and Chagas disease infection, treatment with meglumine antimoniate would be beneficial to modulate patient’s immune responses toward a more protective profile to both diseases.
